# Vestibular Function and Postural Control in Children with Autism Spectrum Disorder

**DOI:** 10.3390/jcm13175323

**Published:** 2024-09-09

**Authors:** Donella Chisari, Jessica Vitkovic, Ross Clark, Gary Rance

**Affiliations:** 1 Department of Audiology and Speech Pathology, The University of Melbourne, Parkville, VIC 3010, Australiagrance@unimelb.edu.au (G.R.); 2 Dizzyology Australia Limited, Melbourne, VIC 3149, Australia; 3 School of Health and Behavioural Sciences, University of the Sunshine Coast, Sunshine Coast, QLD 4556, Australia

**Keywords:** autism spectrum disorder, vestibular, postural control, postural sway, functional balance

## Abstract

**Background:** Postural control deficits have been documented in children with autism spectrum disorder (ASD), yet vestibular system contributions to postural control have not been widely considered. The purpose of this study is to explore the relationship between functional balance, postural sway, and vestibular function in children with ASD. **Methods:** Ten children with a confirmed diagnosis of ASD according to DSM-V guidelines along with ten children with no known neurodevelopmental or motor delays participated in the study. Bruininks–Oseretsky Test of Motor Proficiency and the Paediatric Balance Scale measured functional balance ability, and postural sway was measured using static posturography with modified sensory inputs. Peripheral vestibular function was measured using cervical vestibular evoked myogenic potentials and video head impulse testing. Correlations between measures were performed. **Results:** When visual cues were removed, children with ASD demonstrated larger path velocities indicative of reduced postural control, and different patterns of postural sway. Functional balance was correlated with path velocities for conditions where sensory information was modified. No differences in peripheral vestibular function were noted between groups, and functional balance was not correlated with vestibular function. **Conclusions:** Findings suggest that while peripheral vestibular function is similar between groups, postural control differences in children with ASD remain, particularly for conditions where sensory information is modified. Furthermore, demonstrated patterns of postural sway suggest sensory system integration is less developed in children with ASD. These findings highlight the importance of utilising a range of clinical tools to quantify balance ability and consideration of postural control measures to inform intervention.

## 1. Introduction

Autism spectrum disorder is a neurodevelopmental condition with an estimated prevalence of 1 in 40 [1,2,3], characterised by impairments in communication, social reciprocity, and repetitive behaviours [4]. Differences in motor behaviour have also been documented in children with ASD [5] including postural stability deficits [6], gross motor skill delays [7], and functional balance abnormalities.

For children with ASD, evidence suggests that underconnectivity between central mechanisms of the cerebellum or general neural circuitry affects postural control [8]. This is particularly evident for more complex motor tasks or when sensory information is modified [8]. Yet the precise mechanisms of postural stability deficits in children with ASD remain unclear.

Functional balance measures typically provide an indication of motor skills including fine manual control, body coordination, and balance [9]. Static posturography is one way of assessing postural control; a force plate is used to measure the postural sway of an individual over a specified time period. Additionally, static posturography can reflect sensory system contributions (visual, somatosensory, vestibular) and predominance in various sensory conditions [7,10,11]. For people with ASD, when sensory information is modified via methods such as standing on foam or closing eyes, postural sway is more pronounced [7,10,11]. Some authors have noted deficits across all sensory conditions [6,8], whereas others have only found deficits in more challenging sensory environments [12,13]. Furthermore, these attributes have largely been explored in adults and are yet to be thoroughly explored in children. Evaluating postural control in children is complicated by the fact that individual sensory system contributions to postural control vary across childhood [14].

Postural sway has typically been measured using centre of pressure (CoP) measurements, including path length, path velocity, and area. However, breaking down CoP measurements into discrete frequency bands based on the speed of postural sway (referred to as discrete wavelet transform [DWT]) analyses can provide a more granular interpretation of the movement and include an indicator of relative physiological contributions to postural control [15,16]. It is thought that certain frequency components of the CoP data represent different sensory mechanisms: somatosensory (0.5–1.0 Hz), vestibular (0.1–0.5 Hz), and vision (<0.1 Hz) [15,17]. Discrete wavelet transform analyses have provided more useful information than regular CoP data in clinical groups including adults with Parkinson’s disease [18] or chronic neck pain [19], as well as children with mild traumatic brain injury [20]. More recently, DWT has been explored in typically developing children [16].

Vestibular function is fundamental to balance, and in children, peripheral vestibular assessment serves an important role in understanding the mechanisms of motor delay and management of balance difficulties. A range of clinical vestibular assessment tools can be used to quantify vestibular function in children, including assessment of the semicircular canals (SCC) [21,22] and otolith organs: the utricle and saccule [23,24,25,26]. Additionally, the role of vestibular function in standing balance relies on central processing mechanisms to assist with providing appropriate motor outputs. Though studies have demonstrated that children with ASD have deficits in motor skill development (and this relates to postural control [7]), the relationship between vestibular function in children with ASD and postural control has not been directly evaluated. Furthermore, understanding movement patterns by exploring discrete frequency bands has not been considered in children with ASD and may provide further insight into the physiologic mechanisms underpinning movement patterns during static balance. These assessments may provide a more cohesive overview of balance ability in children with ASD and be useful for diagnosis and management of motor behaviour deficits.

The primary study aim was to explore vestibular function and postural control in children with ASD. We hypothesised that clinical assessments of vestibular function would be similar between children with ASD and those with neurotypical development, yet postural sway measures would be larger for children with ASD across conditions where sensory information is modified. Furthermore, we hypothesised that a relationship exists between motor skills and postural sway measures, including path velocity and the fast-moving component of the CoP trace.

## 2. Materials and Methods

### 2.1. Participants

Twenty children between 5 and 12 years of age were recruited via audiologists, speech pathologists, and paediatricians in clinical settings across Melbourne, Australia. Ethical approval was granted (approval number 17-1348H), and procedures were conducted according to tenets of the Declaration of Helsinki. Guardians provided written consent, and verbal assent was obtained from each child.

Children were age matched within three months and met the following criteria: children in the ASD group (*n* = 10) had a confirmed diagnosis of ASD according to DSM-V guidelines [4]; children in the neurotypical (NT) group (*n* = 10) had no known neurodevelopmental or motor delays. Participants required normal sound detection and middle ear function at the time of assessment, as both factors can influence peripheral vestibular results [27,28]. All participants understood simple instructions.

### 2.2. Audiometry and Immittance

Behavioural thresholds for pure tone audiometry were established at octave frequencies between 250 Hz and 8 kHz using an Interacoustics Affinity PC based audiometer (Interacoustics, Middelfart, Denmark; version 2.0). Tympanometry and ipsilateral acoustic reflex assessment determined middle ear status (GSI Tympstar, Grason-Stadler Instruments, Eden Prairie, MN, USA) [29].

### 2.3. Semicircular Canal Assessment

The video head impulse test (vHIT) was performed to assess high-frequency semicircular canal (SCC) function (GN Otometrics ICS Impulse System, GN Otometrics, Taastrup, Denmark). Overall VOR gain was determined by comparing slow phase eye velocity to the head impulse velocity. Task administration was based on standard clinical procedures [30]. Coplanar SCC pairs were assessed: lateral, right anterior/left posterior SCC (RALP), and left anterior/right posterior SCC (LARP). At least ten head impulses were performed for each condition. Head impulse peak velocity ranged from 150–250 degrees/s and magnitude ranged from 15–20 degrees from centre. GN Otometrics statistical software (version 4.1) was used to process and analyse data. Prior to data analysis, an experienced audiologist visually inspected each trial and removed artefacts caused by pupil tracking difficulties or goggle slippage.

### 2.4. Otolith Assessment

Cervical VEMP (cVEMP) testing was performed using air-conducted stimuli (500 Hz pure tone, stimulus rate 5.1 Hz, presented at 90 dBHL) to measure saccule function (Natus Bio-Logic NavPro, Natus, Pleasanton, CA, USA; Bio-Logic AEP software version 7.0.0). Electrophysiological responses were recorded using a four electrode-montage setup: an inverting electrode placed on each sternocleidomastoid muscle belly, a common electrode placed at Nz, and a non-inverting electrode placed at the sternoclavicular junction. Standard clinical procedures were used to elicit the cVEMP [31], and post-stimulus rectification was applied to the waveforms. Stimuli were presented alternately between ears to minimise fatigue effects on responses, and for each condition, two traces were conducted for repeatability. Established labelling conventions were used to mark cVEMP latencies (P13, N23) [31,32] and response amplitudes calculated.

### 2.5. Functional Balance

Two subtests from the Bruininks–Oseretsky Test of Motor Proficiency 2nd Edition (BOT-2) [33], bilateral coordination and balance, were administered to all participants. Standard scores and percentile ranks were determined using sex-specific norms, and an overall composite body coordination score was calculated.

The Paediatric Balance Scale (PBS) a supplementary assessment used to quantify motor performance [34] included fourteen motor tasks subjectively rated by the examiner.

### 2.6. Standing Balance

Computerised static posturography was conducted using the Nintendo Wii Balance Board (WBB), connected to a laptop using Bluetooth Classic, with customised LabVIEW 2009 (Version 8.5, National Instruments, Austin, TX, USA) software to enable data recording at the native frequency (≈40 Hz). Device calibration and signal processing were implemented according to previous protocols [35,36].

Postural sway was objectively measured using centre of pressure (CoP) information in four sensory conditions: standing with eyes open on a firm surface (EO), eyes closed on a firm surface (EC), eyes open on a foam surface (FEO), and eyes closed on a foam surface (FEC). For two conditions, a 5 cm foam cushion fit completely over the platform.

The assessment was conducted barefoot, and a practice trial was performed to ensure task understanding. For each condition, three 30 s trials were completed, with the median score used for further statistical analyses. Height and weight characteristics were obtained following postural sway assessment.

Centre of pressure (CoP) measures included path velocity (cm/s) and DWT analyses, where the CoP trace is broken down into discrete frequency bands. The frequency bands correspond to ranges from very slow moving to rapid components of the CoP data and included <0.10 Hz (ultra-low), 0.10–0.39 Hz (very low), 0.39–1.56 Hz (low), and 1.56–6.25 Hz (moderate) frequency bands using the methods described previously [37]. Centre of pressure was analysed across the four standing conditions in the anterior/posterior plane, given the task was conducted for bipedal stance. The Romberg quotient was also calculated (EC/EO), with a score > 1 indicating larger sway with eyes closed [20].

### 2.7. Statistical Analysis

All data except for the PBS were normally distributed based on statistical analysis. Outcome measures consisted of VOR gain (for vHIT), latencies and amplitudes (for VEMP), BOT balance scale score, BOT percentile ranks, BOT body coordination scale core, PBS overall score, and CoP data (path velocity, DWT for individual frequency bands). Normally distributed outcome measures were assessed using general linear modelling and independent *t*-tests. Mann–Whitney U was used to compare PBS data. Participant height and weight were considered as covariates for CoP analyses. Post hoc Tukey analyses were set at a significance level of *p* < 0.05. Effect sizes were calculated using Hedge’s g [38]. Pairwise Pearson correlations explored the association between functional balance, postural sway, and vestibular results across all children.

## 3. Results

Every child completed the assessments of standing balance and peripheral vestibular function. One child from the ASD group did not complete the PBS and body coordination subtest of BOT. Left and right responses for tests of peripheral vestibular function were not significantly different and therefore averaged for statistical analysis. Group demographics are presented in Table 1 and Table 2.

Overall functional balance measures were different from each other: children with ASD had lower PBS scores when adjusted for ties (NT Mdn [IQR] = 54 [1] vs. ASD Mdn [IQR] = 53 [2], *p* = 0.044), but BOT percentile ranks were similar (NT mean [SD] = 58.0 [24.9] vs. ASD mean [SD] = 42.8 [24.8], *p* = 0.201). However, exploration of the BOT subtests demonstrated that children with ASD showed significantly lower balance scores (NT mean [SD] = 15.4 [3.69] vs. ASD mean [SD] = 11.9 [3.18], *p* = 0.036), but no differences were observed between groups for body coordination (NT mean [SD] = 17.30 [3.27] vs. ASD mean [SD] = 16.33 [3.32], *p* = 0.532).

While functional balance measures were different between children with neurotypical development and children with ASD, peripheral vestibular function was similar, with findings reported in Table 3. No statistically significant differences in cVEMP latencies or amplitudes were observed inter-group, indicating that both groups had similar saccule function. vHIT findings showed comparable VOR gain across conditions, suggesting that high frequency semicircular canal function was similar. These findings demonstrate that peripheral vestibular function is normal in children with ASD.

**Figure 1:**
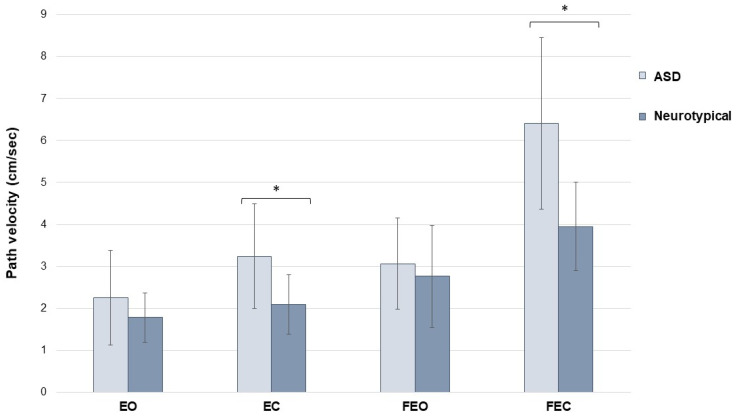
Mean path velocity for children with and without ASD across eyes open (EO), eyes closed (EC), and those conditions repeated on foam (FEO and FEC, respectively). Children with ASD possessed significantly reduced postural stability (indicated by increased path velocities) when visual information was removed (eyes closed conditions on firm and foam surfaces). Error bars represent standard deviations (SD), and asterisks denote *p* < 0.05.

When measuring postural stability, children with ASD demonstrated significantly faster path velocities when visual cues were removed (EC: NT mean [SD] = 2.09 [0.71] vs. ASD mean [SD] = 3.24 [1.25], *p* = 0.024; FEC: NT mean [SD] = 3.95 [1.06] vs. ASD mean [SD] = 6.41 [2.04], *p* = 0.005). Figure 1 illustrates path velocities between groups; faster path velocities are indicative of poorer postural control. In contrast, path velocity was similar between groups when visual information was available (EO: NT mean [SD] = 1.78 [0.59] vs. ASD mean [SD] = 2.25 [1.13], *p* = 0.253; FEO: NT mean [SD] = 2.76 [1.22] vs. ASD mean [SD] = 3.06 [1.09], *p* = 0.577). This also corresponded to a significantly larger Romberg ratio in children with ASD (NT mean [SD] = 1.12 [0.23], ASD mean [SD] = 1.58 [0.42], *p* = 0.009).

Post hoc comparisons of DWT analyses showed that children with ASD had significantly larger contributions in moderate and low DWT frequency bands when visual information was removed (EC, FEC) and somatosensory information was modified (FEC). No significant differences between groups across EO and FEO conditions were noted, as demonstrated in Table 4.

Postural stability was correlated with functional balance. Significant, negative correlations were observed with standing balance and functional balance measures, particularly where visual and somatosensory input was modified (FEC condition). Lower PBS scores indicative of poorer functional balance were associated with larger path velocities (r(19) = −0.799, *p* < 0.001) and larger contributions from the faster components of CoP data (moderate DWT: r(19) = −0.634, *p* = 0.004; low DWT: r(19) = −0.737, *p* < 0.001). Lower balance scale scores from BOT indicative of poorer balance function were also associated with larger path velocities (r(20) = −0.597, *p =* 0.005) and larger contributions from DWT moderate and DWT low frequency bands (moderate DWT: r(20) = −0.585, *p* = 0.007; low DWT: r(19) = −0.453, *p* < 0.045). Larger path velocities from the moderate DWT frequency band were correlated with lower body coordination scores and overall percentile ranks (BOT body coordination: r(19) = −0.497, *p =* 0.030; BOT percentile rank: r(19) = −0.504, *p =* 0.028). No correlations were observed between vestibular function (which was typically normal) and functional balance.

## 4. Discussion

This study quantified peripheral vestibular function, motor proficiency, and standing balance ability in children with ASD. Children with ASD show compromised functional balance, which in turn has repercussions for balance during daily activities. Despite normal peripheral vestibular function, reduced postural stability was observed when visual inputs were compromised, suggesting greater reliance on visual information. Furthermore, children with ASD showed greater postural sway for the faster moving components of the CoP data, illustrating that sensory system integration may be different for children with ASD when compared to children with neurotypical development.

Functional balance and postural stability can be influenced by the vestibular system, as demonstrated in other groups of individuals with documented peripheral vestibular dysfunction [39,40,41]. In this study, peripheral vestibular function is normal in children with ASD and therefore is not the primary factor for differences in functional balance and overall postural control. The cerebellum and vestibular system are intricately linked [42]. Measures of peripheral vestibular function provide an overall representation of several elements: the integrity of the peripheral end organs, their corresponding neural reflex pathways, and, to a lesser extent, the structures involved in producing the output. In addition to detecting motion, the vestibular system’s contributions to postural control are predominately for head and body stabilisation [42,43,44]. In instances where proprioceptive and visual cues are reduced, reliance on the vestibular system and vestibular reflexes increases. Therefore, given that peripheral vestibular function is normal in children with ASD, the differences observed with postural stability and functional balance suggest that the central processing mechanisms may be underdeveloped, which in turn may influence the processing of sensory information during standing balance [45].

Postural stability was similar between groups when eyes were open but different when eyes were closed. For children with ASD, increased postural sway, indicative of reduced postural stability, was most notable once visual information was removed. This finding suggests that children with ASD have greater reliance on visual cues to maintain balance, and to an extent, may indicate developmental differences in sensory system integration [11,12]. Visual system predominance during standing balance is dependent on the nature of the balance task and whether movement is involved. For younger children, the visual system is the main contributor to balance control due to insufficient multimodal sensory integration [46,47], even with static balance tasks, but there is evidence to suggest that there is less reliance on vision with increasing age. Children with ASD in this study showed a similar pattern of visual predominance, which may suggest immaturity of sensory system integration.

Greater sensory integration is required for more complex standing conditions. In this study, the most difficult standing balance condition (FEC) was more challenging for children with ASD. In particular, children with ASD showed greater postural sway for the fast-moving components of the CoP data (moderate and low frequency bands) when visual and somatosensory information were modified. While these frequency bands are thought to correlate with somatosensory and cerebellar inputs, this may be due to greater difficulties with postural control integration as demonstrated by larger amounts of sway. Furthermore, fast-moving components of postural sway were negatively correlated with functional balance; those with lower PBS and BOT balance scores exhibited greater amounts of fast-moving components in their postural stability compared to children who were more stable. It is known visual predominance for postural control occurs in younger children, and there is a readjustment of sensory system predominance as the vestibular and somatosensory systems become better integrated in the overall process of postural control [48,49]. These findings suggest that sensory integration processes are underdeveloped in children with ASD, thereby corresponding to poorer functional performance.

The cerebellum is considered the central mechanism for multimodal sensory integration and serves an important role in refining and executing vestibular mediated reflexes and motor outputs, or motor coordination [50]. Sensory system information is received by the central processing mechanisms, where the information is processed and results in a motor or reflexive output. Previous studies have identified differences in the cerebellum in ASD related to reduced Purkinjie cells [51,52], but also decreased functional connectivity between the connective structures involved in integrative processes within the brain [53,54,55]. These differences could be linked to the observations noted in this study; for children with ASD, greater reliance on visual information was observed, and for more complex standing balance conditions, the fast-moving components of postural sway were predominant. These findings were in the context of normal vestibular input during standing balance conditions. Thus, these findings may unveil difficulties with integrating more complex sensory information rather than sensory impairment per se.

Clinically, vestibular function was similar between children with ASD and neurotypical development. For children with ASD presenting with balance concerns, formal vestibular function assessment would still be useful to rule out an additional peripheral vestibular disorder. In instances where no peripheral deficits are identified, the balance concerns may be related to sensory integration issues based on deficits identified in this study. If a peripheral vestibular deficit is identified, these findings would be useful to guide management and ensure that deficits are holistically addressed via the inclusion of vestibular rehabilitation.

## 5. Study Limitations

There are several study limitations. Firstly, the vestibular test battery did not include measures of all peripheral vestibular organs. As utricle function was not assessed, there is the possibility that some participants had utricular dysfunction, potentially influencing results. However, the likelihood of utricular dysfunction in the context of other normal vestibular findings is small. Secondly, the small sample size could not provide gender comparisons, limited generalisation of the findings, and reduced statistical power. Despite the small sample size, large effect sizes were noted between groups on standing balance measures, and moderate to strong correlations were noted on functional balance measures. Finally, only children between 5 and 12 years old were recruited in this study, and therefore the findings cannot be applied to other age groups. Further research is required to understand if the deficits identified in this study are resolved throughout childhood or continue to adulthood.

## 6. Conclusions

Postural control deficits in children with ASD may be related to a maturational deficit in sensory system integration or related to cerebellar underconnectivity. Further research is required to better understand the relationship between postural control, vestibular function, and sensory system reweighting in children with ASD. Utilising a range of clinical assessments for children with balance or motor development concerns can reveal potential functional consequences, and aid in addressing balance concerns more comprehensively.

This study also highlights the importance of considering alternative postural control measures, such as fast-moving components of postural sway data. This movement analysis of postural control may be a more comprehensive way to quantify postural stability in children with ASD and provide the clinician with a broader understanding of underdeveloped areas for postural control. In turn, these measures may supplement intervention options so therapists can provide specific exercises to address the deficits identified.

## Figures and Tables

**Table 1:** Overall group demographics.

						Audiometry	
Group	*N*	Male:Female Ratio	Age (years)	Height (cm)	Weight (kg)	4FHLA Left (dBHL)	4FHLA Right (dBHL)
NT	10	6:4	7.77 (5.92–9.92)	130.45 (10.11)	30.08 (11.6)	6.38 (5.91)	8.25 (1.55)
ASD	10	7:3	7.62 (5.42–9.92)	132.20 (13.89)	31.86 (6.51)	11.25 (2.82)	10.75 (2.37)

Mean values (with range or SD) reported for each demographic; NT: neurotypical; ASD: autism spectrum disorder; 4FHLA: four frequency hearing level average across 500 Hz, 1 kHz, 2 kHz and 4 kHz; dBHL: decibels hearing level.

**Table 2:** Participant demographics for assessments used in ASD diagnosis.

Participant	Gender	Age at Assessment (years)	Assessments Used in ASD Diagnosis
ASD1	F	5.42	DSM-V, CELF-P, WPPSI-IV
ASD2	M	9.33	DSM-V, WISC-V, CELF-4
ASD3	F	7.50	DSM-V, WISC-V, CELF-5
ASD4	M	6.92	DSM-V, WPPSI-IV, CELF-P2, DBC, ADOS
ASD5	M	7.17	DSM-V, CELF-5, ADOS, Mullen, ADI-R
ASD6	M	8.67	DSM-V, PEP-3, ABC, CARS, CELF-5
ASD7	M	8.25	DSM-V, CELF-4
ASD8	M	6.92	DSM-V, WPPSI-IV, ADOS, ABC, CELF-4
ASD9	F	6.08	WPPSI-IV, ADOS, CELF-5
ASD10	M	9.92	DSM-V, CELF-5, ADOS, VABS, WNV

ABC: Autism Behaviour Checklist; ADI-R: Autism Diagnostic Interview-Revised; ADOS: Autism Diagnostic Observation Scale; CARS: Childhood Autism Rating Scale; CELF-P, CELF-4, CELF-5: Clinical Evaluation of Language Fundamentals (Preschool, Editions 4, 5); DBC: Developmental Behaviour Checklist; DSM-V: Diagnostic and Statistical Manual of Mental Disorders (Edition V); PEP-3: The Psychoeducational Profile (Version 3); VABS: Vineland Adaptive Behaviour Scales (Version 3); WISC: Wechsler Intelligence Scales for Children (Version V); WPPSI: Wechsler Pre-School and Primary Scale of Intelligence (Version IV); WNV: Wechsler Nonverbal Scale of Ability.

**Table 3:** Group comparisons for peripheral vestibular function measures of cVEMP and vHIT measures of.

		ASD		NT			
cVEMP		Mean (SD)	Range	Mean (SD)	Range	ES	*p*
Latencies (ms)	P13	13.90 (1.50)	11.37–16.65	13.42 (1.32)	10.44–15.75	0.340	0.290
	N23	19.73 (1.68)	15.54–21.54	19.76 (1.37)	17.5–22.1	0.020	0.942
Amplitude (µV)	P13-N23	12.59 (4.72)	3.90–20.28	9.97 (3.87)	4.73–21.67	0.607	0.063
vHIT							
	HSCC	0.98 (0.09)	0.83–1.2	0.93 (0.05)	0.85–1.03	0.687	0.051
VOR gain	PSCC	0.95 (0.09)	0.75–1.05	0.96 (0.20)	0.71–1.34	0.065	0.828
	ASCC	0.97 (0.13)	0.70–1.16	0.94 (0.14)	0.76–1.22	0.222	0.578

ASD: autism spectrum disorder; NT: neurotypical; cVEMP: cervical vestibular evoked myogenic potentials; P13: P13 latency; N23: N23 latency, P13-N23: peak to peak cVEMP amplitude; vHIT: video head impulse test; VOR: vestibulo-ocular reflex; HSCC: horizontal semicircular canal; PSCC: posterior semicircular canal; ASCC: anterior semicircular canal; ES: effect size (calculated using Hedges g).

**Table 4:** Between-group comparisons for DWT bandwidth velocity across the four frequency bands and four standing balance conditions. Outcome measures reported as mean (SD).

Standing Balance Condition		EO				EC				FEO				FEC			
		ASD	NT	ES	*p*	ASD	NT	ES	*p*	ASD	NT	ES	*p*	ASD	NT	ES	*p*
DWT Bandwidth Velocity (cm/s)	Ultra-low	0.13 (0.08)	0.09 (0.37)	0.149	0.182	0.14 (0.04)	0.11 (0.06)	0.588	0.119	0.10 (0.04)	0.12 (0.05)	0.441	0.425	0.17 (0.03)	0.14 (0.05)	0.728	0.200
	Very Low	0.39 (0.29)	0.30 (0.09)	0.419	0.365	0.54 (0.26)	0.41 (0.15)	0.612	0.087	0.57 (0.32)	0.54 (0.22)	0.109	0.808	1.04 (0.40)	0.92 (0.47)	0.275	0.573
	Low	0.96 (0.70)	0.79 (0.23)	0.326	0.320	1.51 (0.72)	1.08 (0.33)	0.768	0.018 *	1.33 (0.39)	1.33 (0.60)	0.506	0.817	2.92 (1.15)	2.03 (0.62)	0.963	0.013 *
	Moderate	0.86 (0.42)	0.79 (0.25)	0.203	0.480	1.43 (0.53)	1.02 (0.37)	0.897	0.019 *	1.35 (0.73)	1.25 (0.56)	0.651	0.646	3.16 (1.21)	2.07 (0.81)	1.06	0.011 *

* *p* < 0.05 adjusted using Tukey post hoc; DWT: discrete wavelet transform; cm/s: centimetres per second; EO: eyes open; EC: eyes closed; FEO: foam eyes open; FEC: foam eyes closed; ES: effect size (Hedges g).

## Data Availability

The original contributions presented in the study are included in the article, further inquiries can be directed to the corresponding authors.
